# The Biological Object Notation (BON): a structured file format for biological data

**DOI:** 10.1038/s41598-018-28016-6

**Published:** 2018-06-25

**Authors:** Jan P. Buchmann, Mathieu Fourment, Edward C. Holmes

**Affiliations:** 1 Marie Bashir Institute for Infectious Diseases and Biosecurity, Charles Perkins Centre, School of Life and Environmental Sciences and Sydney Medical School, the University of Sydney, Sydney, New South Wales 2006 Australia; 2 ithree Institute, University of Technology Sydney, Ultimo, New South Wales 2007 Australia

## Abstract

The large size and high complexity of biological data can represent a major methodological challenge for the analysis and exchange of data sets between computers and applications. There has also been a substantial increase in the amount of metadata associated with biological data sets, which is being increasingly incorporated into existing data formats. Despite the existence of structured formats based on XML, biological data sets are mainly formatted using unstructured file formats, and the incorporation of metadata results in increasingly complex parsing routines such that they become more error prone. To overcome these problems, we present the “biological object notation” (BON) format, a new way to exchange and parse nearly all biological data sets more efficiently and with less error than other currently available formats. Based on JavaScript Object Notation (JSON), BON simplifies parsing by clearly separating the biological data from its metadata and reduces complexity compared to XML based formats. The ability to selectively compress data up to 87% compared to other file formats and the reduced complexity results in improved transfer times and less error prone applications.

## Introduction

Biological data, which includes, but is not limited to molecular sequences, annotations and phylogenetic trees, are still predominantly exchanged as flat files or in line-based formats despite the existence of more structured file notations that are better suited to complex data. Here, we propose an improved format for biological data that overcomes many of the limitations associated with flat files. This new “biological notation” (BON) format is based on the JSON notation and can support all data types required in biology. We do not propose a new standard, but a rather new structured and lean file format for modern molecular biology data sets and their exchange. We implemented BON as a Python library that can be used in pipelines and as basis for individual adjustments.

Since the emergence of the first successful format for biological sequences – the FASTA format in 1985^1^ – different formats for a variety of biological data types have been developed, such as molecular sequences (FASTQ^2^), phylogenetic trees (Newick^2^, NEXUS^3^), and sequence alignments (Stockholm^4^, BAM^5^). These formats are all flat files or block-based and hence inflexible to modification, complicating the addition of additional information. Further, each format requires a different parser that must be developed and maintained. Although unstructured flat files are commonly used for biological data sets, no new approaches have been proposed to simplify the exchange of increasingly complex sequence data sets.

**Figure 1:**
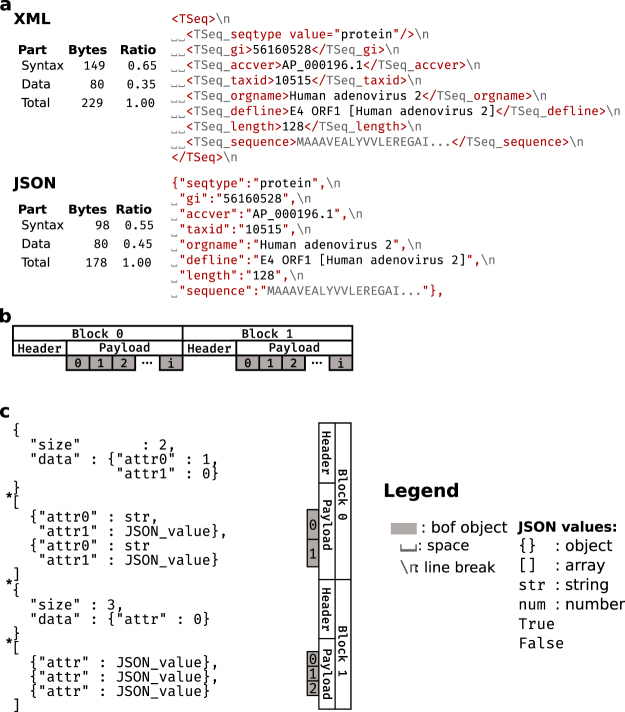
Basic description of BON. (**a**) TinySeq XML and an equivalent JSON notation to demonstrate the ratio of clutter between these syntaxes. The tables on the left indicate the size of the attributes and the XML tags or JSON keys, respectively. Sizes are given in bytes. Only those parts shown in red are included in the size calculations. (**b**) Schematic diagram of the BON format indicating its major components. (**c**) Basic BON example in JSON syntax. Stars indicate positions where header and payload neighbour using non-valid JSON syntax. The JSON snippet in (**a**) is an example of a possible payload item and the sequence attribute is an obvious candidate for data compression.

The “Extensible Markup Language” (XML) has been adopted as a mark-up language for the exchange of structured biological data, including TinySeq (NCBI), INSDSEQ (http://www.insdc.org/documents), NeXML^6^ or phyloXML^7^. However, XML is complex compared to JSON (Supplementary Material Notes 1,2) and requires at least four parts: (i) the document type definition schema language (DTD, https://www.w3.org/TR/xmlschema-1/), (ii) the XML Schema Definition schema language (XSD, https://www.w3.org/TR/xmlschema11-1/), (iii) the Extensible Stylesheet Language Transformations transformation language (XSLT, https://www.w3.org/TR/xslt20/), and (iv) the XML Query (XQuery) and XML Path Language (XPath, https://www.w3.org/TR/xpath-datamodel-31/). Recently, the World Wide Web Consortium proposed an “Efficient XML interchange format” (EXI^8^). While the EXI specifications indicate improved transfer times due to an optimized binary form of XML, the underlying structure is still XML. XML documents tend to have a high degree of “noise” or “clutter”, as the syntax can use more space than the information itself (Fig. 1a). We define clutter as any part of the transferred data not related to meta- or biological data, such as file specific syntax like XML tags or JSON syntax. In contrast, the JSON notation can encode the same data as XML, but with cleaner syntax and less clutter.

Parsing errors can occur when parts of the data are wrongly classified. Metadata can and is increasingly incorporated into the existing flat file formats, for example FASTA, by extending the header line and using a specific character as delimiter. These extensions are highly variable as they depend on the author of the file (Supplementary Notes 3). Therefore, if such metadata is present it has to be carefully checked since such extension are not standardized. Implementing such checks and the corresponding corrections subsequently increase the complexity of the parser, thereby increasing the change to introduce additional errors. The reduced complexity of the JSON syntax makes it possible to write parsers with simpler code and ultimately fewer errors. These are important factors, considering the steadily increasing size and complexity of biological data that must be transferred between computers and applications.

Biological data repositories such as the European Molecular Biology Laboratory (EMBL, https://www.embl.org/) and the National Center for Biotechnology Information (NCBI, https://www.ncbi.nlm.nih.gov/) offer JSON as a data format within several “Representational state transfer” (REST) application programming interfaces (APIs). For example, NCBI’s Entrez utility or REST API only export biological data in FASTA and XML formats, although other information is available in JSON^9^. In biology, JSON is mainly used to store or exchange application related data that can include biological data, such as alignments^10^ or protein sequences^11^. Another use of JSON is to combine search results^12^ or represent XML schemes^13^. To date, however, the JSON syntax has not been used to describe biological data independent of an application or schematic dependency.

We can only speculate why JSON has not been yet widely adopted for biological data. It is possible that the prevalence of XML established a *de facto* standard that works for most biologists. However, the increased data complexity in biology will require biologists to write their own tools, and hence parsers, specifically tailored to their research. Importantly, JSON notation represents data the same way as used in programming, while XML requires a transformation step, adding unnecessary complexity. The existence of JSON parsers in almost all programming languages facilitates adapting existing and creating new pipelines. With the large amount of data now common in biology, human readability is of no concern and efficiently inspecting data must be done by specific tools. Herein, we use the JSON syntax in our new biological notation format to facilitate the inspection and exchange of structured biological data with the following properties.

### Reduced parsing complexity

**Figure 2:**
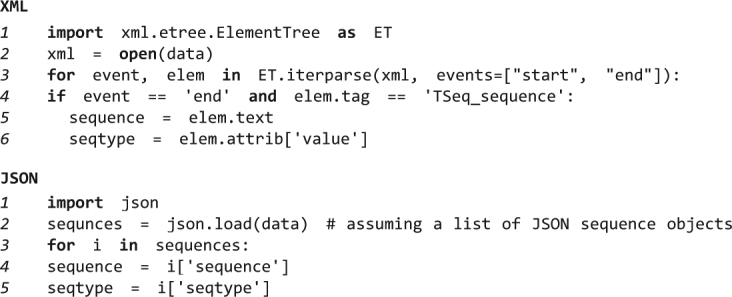
A simplified example illustrating the differences in parsing XML and JSON syntax in Python. Numbers in italic indicate line numbers for each example. Python syntax is indicated in bold. The XML syntax requires more checks since XML tags can open or close (‘start’ or ‘end’ event, respectively) and properties can be indicated as tag attributes, here ‘seqtype’ as encountered in NCBI’s TinySeq XML. The JSON syntax facilitates data access and therefore reduces parsing complexity. Checks for the existence of the expected data have been omitted for simplicity.

The JSON syntax describes only two structures; ordered list and named/value pairs. These structures can describe virtually all biological data sets while retaining low parsing complexity, for example because additional checks like those for attribute values in XML tags are omitted. Complex data can still be described, such as the use of nested structures, but the structure limitation simplifies writing of the corresponding parsers. JSON can be therefore considered a very lean XML^14^. An example comparing parsing complexity is given in Fig. 2.

### Scheme independent

There are no fixed keywords, only a few rules on how to describe and handle data. This allows adjustments to be made for the majority, if not all, biological data sets. The keywords are defined by the application authors or maintainers, and often explained in the source files or accompanying documentation. The simple rule set and the close link to the corresponding data set, not a specific scheme, make the format more decentralized and therefore resilient to change.

### Selective compression

Biological data is steadily increasing in size and complexity. To facilitate data exchange, for example via streams, large chunks of the data set can be compressed while metadata is accessible (but can be compressed as well). This allows the user to screen and filter the data set while receiving it, thereby avoiding temporary files.

### Application agnostic

The data can be read by any application able to parse JSON and does not store application specific information. Ensuring the implementation of standardized compression algorithms that are available as standard libraries for the majority of programming languages guarantees this condition.

## Results

We devised a new file format for biological data, denoted BON, and tested it on both molecular sequence and phylogenetic data. Nucleotide and protein sequences use almost all available characters, have different lengths and contain metadata. They therefore represent good benchmarks to measure the extent of the compression achieved by BON. We designed BON with the ability to store compressed data since the cost of the transfer, such as downloading or streaming data between two applications, is higher than compressing or decompressing data. To demonstrate the versatility of BON to handle non-sequence data we apply it to phylogenetic data. In addition, we wrote proof-of-concept tools using our BON implementation to convert NCBI’s TinySeq XML, FASTQ, and NeXML formats into BON.

### BON structure

The underlying basic structure of BON consists of a header (a JSON object), followed by a JSON array containing JSON objects that is designated as the “payload” (Fig. 1b). A header and the corresponding payload constitute a BON block that can be concatenated (Fig. 1b,c). The payload follows directly after the header, and a new BON block can follow immediately after the payload (Fig. 1b,c). The immediate sequence of header and payload, indicated as“}[”, is not valid JSON syntax. However, it is a unique signal facilitating parsing and error checking. Due to the uniqueness of this signal, header and payload can be separated using command line tools, e.g. “sed ‘s/\}\[/\n/g’”. Similarly, the signal “]{” indicates a new BON in a stream. Therefore, BON as a whole is not valid JSON, but consist of two parts each of which are valid JSON.

The BON header is a JSON object and contains two mandatory attributes: “size” and “data” (Fig. 1c). The “size” attribute indicates the number of data objects in the corresponding payload, excluding any nested objects; that is, attributes described as objects (see the section on phylogenetic trees below). The “data” attribute is a JSON object describing the data present in each payload. The key (see figure) represents the data name and the corresponding value indicates if the data is compressed. Compressed data is indicated with 1 and uncompressed data with 0 (Fig. 1b). Other attributes can be added to accommodate different data sets. A biological object is a JSON object within a JSON array (Fig. 1b). It stores the different attributes as key/value pairs in JSON notation, containing such elements as a nucleotide or protein sequence or a phylogenetic tree. BON does not store newlines (‘\n’) which further reduces the parsing complexity. We implemented BON in Python 3 and wrote a basic BON reader to read BON data streams, allowing the integration into existing pipelines (see Methods). Biological objects within a payload can be parsed using specific functions or classes that can be included as libraries. The JSON syntax allows only two structures and in conjunction with the minimal self-description of the expected data present in the header, simpler parsers can be written since it is clearly described how the data can be accessed. Of course, developers must document their specific adaptation, but this is the case for every format. BON has the ability to selectively compress attributes. The name of the attribute is not compressed, but the corresponding value is compressed and encoded as a string. The values can be compressed as a zlib string (https://tools.ietf.org/rfc/rfc1950.txt) encoded in base64 (https://tools.ietf.org/rfc/rfc3548.txt). For a more detailed description refer to the Methods and the available code examples referred therein.

### Sequence data in BON

To assess how well BON handles sequence data we compared it to NCBI’s TinySeq XML format. The latter is encoded using XML and demonstrates the typical additional parsing complexity for an XML file, as the type of sequence is encoded as an XML tag attribute (Fig. 1a). We wrote a TinySeq-to-BON converter (see Methods) and designed a BON equivalent of a TinySeq entry using the same naming scheme (Fig. 1a). To demonstrate the compression capability of BON the sequence data was selectively compressed, i.e. only the string containing the sequence was compressed.

**Figure 3:**
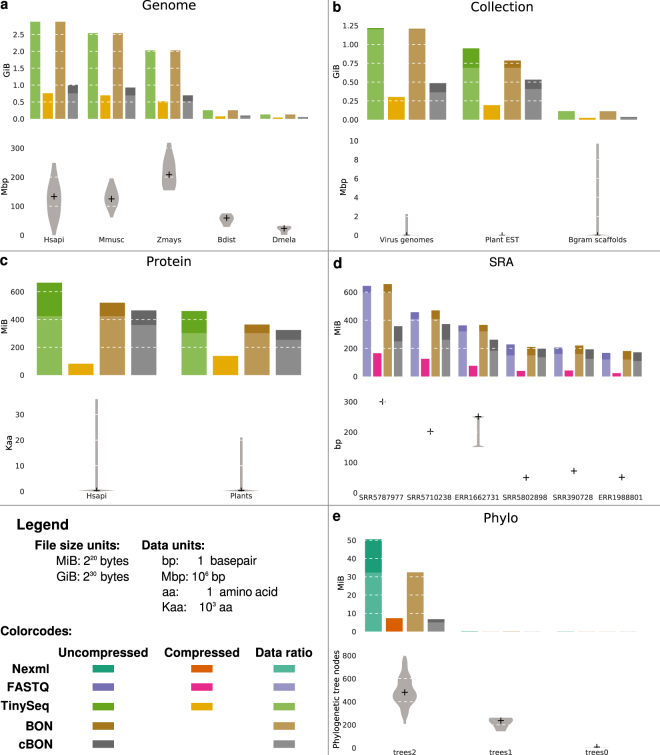
File size and sequence size comparisons for the data sets analysed here. The x-axis depicts the data subset. Upper plots indicate the file size for each subset and the corresponding portion of the data only for uncompressed files, indicated by the lighter colour. The lower violin plots show the corresponding maximum and minimum data sizes analysed within each subset. The “+” indicates the median for the corresponding data set. File sizes and sample data sizes are indicated in the legend. (**a**) The Genome data set compares BON to the TinySeq XML format. Due to their small size the *Escherichia coli* and Saccharomyces cerevisiae genomes are not plotted but given in Supplementary Table 1. The original format is TinySeq XML. (**b**) The Collection data set comparing BON to the TinySeq XML format. (**c**) The SRA data set comparing BON to the FASTQ format. (**d**) The Protein data set comparing BON to the TinySeq XML format. (**e**) The phylogenetic tree (“phylogenetic”) data set comparing BON to the NeXML format.

To demonstrate the utility of BON we analysed two nucleotide sequence data sets; the “Genome” and “Collection” data sets (Fig. 3a; Supplementary Table 2; see Methods). The Genome set contains data subsets with a small number of long sequences, while the Collection data comprises subsets with a greater number of shorter sequences. In the Genome data set, the smallest genome was that from *Escherichia coli* with one sequence of 4.64 Megabasepairs (Mbp) in length. The subset with the greatest number of sequences was the human genome (a total of 25 sequences ranging from 0.01 Mbp to 249 Mbp and totalling ~3,080 Mbp), while the subset with the longest sequence was the *Zea mays* genome (10 sequences between 155 Mbp and 318 Mbp, totalling ~2,180 Mbp). The Collection data set comprised three sub sets, expressed sequence tags (ESTs) from plants (Plant EST), RefSeq virus genomes (i.e. virus genomes), and scaffolds from the *Blumeria graminis* (fungal) genome (Bgram, Supplementary Table 2). The plant EST subset contained the most sequences (1,000,000) ranging between 1 and 4,802 bp and totalling ~544,350 Mbp, while the virus subset contained 66,725 virus genome sequences ranging from 19 bp to 2,243,109 bp and totalling ~1.28 Gigabasepairs (Gbp). The Bgram sub set consists of 6,845 *B. graminis* genome scaffold sequences between 668 bp and 9,686,481 bp, totalling ~119.73 Mbp.

Finally, we also constructed two protein sequence data sets: “Hsapi” with 1,000,000 human protein and “Plant” with 627,778 plant protein sequences (Supplementary Table 3). Sequences in the Hsapi sub set range between 1 amino acid (aa) and 35,991 aa in length, totalling 336,817,042 aa. The Plant sub set sequences range between 1 aa and 21,004 aa, totalling 232,954,947 aa.

The BON equivalent of a TinySeq XML entry contains the same amount of information but is more easily parsed. We compared the TinySeq XML with a corresponding JSON object and identified approximately 10% less clutter in the JSON syntax (Fig. 1a). The TinySeq and uncompressed BON files in the Genome and Collection data sets were almost identical in size, with the exception of the Plant EST subset that was ~18% smaller in BON. However, the compressed BON files were between 43% and 70% smaller in the nucleotide sequence data sets (Fig. 3a,b; Supplementary Table 2). In the protein data sets, the uncompressed BON files were ~20% smaller and the compressed BON files were ~30% smaller compared to the initial TinySeq format (Fig. 3c; Supplementary Table 3).

As another way to demonstrate differences in file sizes we calculated the data to file size ratio: the higher this ratio, the more data and less clutter. In data subsets containing a low number of sequences, such as the Genome data set, this ratio is almost 1 in TinySeq and both the uncompressed and compressed BON file formats. However, with increasing sequence numbers, BON has an improved clutter ratio compared to XML formats (Fig. 3; Supplementary Table 3). Due to the compression, a decrease in the data ratio with increased entries is expected in compressed BON data sets.

We compared the resulting file sizes of the compressed and uncompressed BON to the TinySeq XML file compressed with gzip (see Methods). The gzip compressed TinySeq XML files showed the highest compression, being between 70% and 90% smaller than the original XML file and between 80% and 40% smaller than compressed BON files (Fig. 3, Supplementary Tables 2, 3). In the Genomes data set, all compressed BON files were approximately 25% larger (Fig. 3, Supplementary Table 2). In the subsets in the Collection data set we observed compressed BON files which were between 35% and 40% larger than the corresponding gzipped XML file (Fig. 3, Supplementary Table 2). The biggest difference was observed within the Protein data set, where the gzipped human protein sub set was approximately 90% smaller than the original file and 80% smaller than compressed BON (Fig. 3, Supplementary Table 3). The gzipped Plant subset was approximately 70% smaller than the original XML file and approximately 60% smaller than its compressed BON counterpart (Fig. 3, Supplementary Table 3).

### FASTQ data in BON

Next, to examine how BON compares to a non-structured file format, we converted FASTQ files into BON. FASTQ has emerged as the *de facto* standard for formatting sequence data and allows the storage of both the sequence and the corresponding quality scores. FASTQ sequence archives are flat files and comprise four parts appearing in sequence (as described in Cock^15^), although this is error prone to parse. It stores the sequence in FASTA format, therefore allowing line breaks which must be considered when parsing. Further, the quality score of each base pair position is encoded as ASCII decimal code between 33–126. Since the character “@” (ASCII code 64) indicates a new entry and can also occur at the beginning of the quality string, a new sequence is not unambiguously indicated. This requires additional testing to differentiate between sequence and quality string in FASTQ. In contrast, BON clearly separates these attributes.

We designed the BON equivalent of a FASTQ entry containing the read id, spot id, sequence, quality, and sequence length elements. We then compressed the sequence and quality elements, thereby demonstrating the ability to compress several attributes within BON (Fig. 3d; Supplementary Table 4). For all uncompressed BON files we observed an increase in file size between 1% and 8%. This is expected since we add attribute names not present in the FASTQ specifications (Fig. 3d, Supplementary Table 4). The exception was data set SRR5802898 which was ~8% smaller. However, among compressed BON files, run ERR1988801 was 2% larger, while all other files were between 6% and 44% smaller (Supplementary Table 4). The anomaly in run ERR1988801 was caused by the attribute “readid” which was a sole integer and therefore unusually short.

Similarly to the XML file analysis, we compared compressed BON file sizes to gzipped FASTQ files (see Methods). All gzipped FASTQ files were between 44% and 60% smaller than the original FASTQ files and approximately 30% smaller than all compressed BON files (Fig. 3, Supplementary Table 4).

### Phylogenetic data in BON

To demonstrate the versatility of BON we designed a method to encode phylogenetic trees using the JSON syntax based on NeXML^6^ and which allows the addition of arbitrary metadata (Fig. 3e; Supplementary Table 5). Metadata is especially important in phylogenetic data sets. While the XML format is often used for phylogenetic data, such as in NeXML and phyloXML, adding extra metadata complicates parsing. BON allows the addition of numerous descriptive attributes while compressing the phylogenetic tree.

We treated each BON object as one phylogenetic tree in which the mandatory attribute “tree” contains the phylogenetic tree. The tree itself is encoded in JSON format as two sets: “vertices” and “edges”. This graph structure is similar to the NeXML^6^ format and to GraphML^16^, a general-purpose XML graph format. Each edge has three mandatory attributes: “id”, “source” and “target”. The “source” and “target” attributes contain references to two vertices and the “id” is a unique identifier. Each vertex also contains a unique identifier “id” and a “name” attribute that represents a taxon name or labelled lineage in the context of a phylogenetic tree. If branch lengths are given, nodes contain the attribute “branch length”. Other metadata, such as an estimate of evolutionary rate and its confidence interval can be specified within an edge definition. Importantly, metadata describing the tree that can be investigated while parsing can be added as an attribute to the BON object. This graph structure is flexible as it is can encode bifurcating trees and more complex representations such as ancestral recombination graphs (ARG) that require several additional attributes (e.g. break-point locations in a sequence alignment, or the location if recombination events in a phylogeny).

We assessed the utility of BON by downloading three phylogenetic subsets from Treebase (https://treebase.org/treebase-web/home.html) and then comparing the NeXML format to both uncompressed and compressed BON. The first subset contained 60 trees with 5 to 7 nodes each. The second subset contained the only five trees but with 144 to 261 nodes. The largest subset contained 541 trees with 26,7281 nodes. BON reduced the file size between 30% and 37% compared to NeXML, while the compressed BON files are between 65% and 87% smaller (Fig. 3e; Supplementary Table 5). We compared gzipped NeXML files to BON (see Methods) and observed a very similar compression ratio for gzip and BON (Fig. 3, Supplementary Table 5).

## Discussion

To effectively analyse the ever-increasing amount of biological data being generated it is important that it is efficiently exchanged between both collaborators and computer applications. With BON, we describe a new file format that is tailored to handle these increased data sizes and their associated metadata. Further, it enables adjustments to be made to virtually all biological data set with only a basic knowledge of computer programming.

Not only has the amount of biological data steadily increased, but also its complexity. It is therefore critical to develop efficient ways to store and analyse any associated metadata. The increasing necessity to add biological metadata can be observed in the SRA database at NCBI (https://www.ncbi.nlm.nih.gov/sra), where the length of the sequence is added to the first line of each entry when using the SRA toolkit. Such metadata is especially important in phylogenetic data sets. However, a recent study demonstrated that the widely used Newick format is prone to unclear semantics, which can lead to misinterpretation of the data analysed^17^. BON alleviates these shortcomings by using a less complex approach than XML formats. Importantly, BON has not been designed to be human readable, but to write less error-prone tools to exchange, screen or prepare large data sets for analyses. The JSON notation further supports this approach since it uses structures that are familiar to programmers. BON can encode the same complex data as any XML document due to the ability of nested JSON syntax and data validation can be easily added using checksums as a data field.

Compressed XML and FASTQ files showed the smallest file sizes for all data sets. The biggest factor for the increased file sizes of compressed BON compared to gzipped files is the encoding of the compressed data as base64 string in BON. It introduces an overhead of 33% but allows to transfer binary data as plain text. Another factor influencing the file size is the zlib algorithm. It works more efficiently with repetitive data or when more data can be compressed at once, which is the case for whole files but not in BON. Interestingly, a gzipped version of the human protein subset from the uncompressed BON file is approximately 30% larger than the corresponding gzipped XML file. However, a gzipped uncompressed BON file of the plant protein subset is approximately 5% smaller than a corresponding gzipped XML file. The compression efficiency can be optimized depending the data. The EXI specification is using this fact to obtain better and faster data transfer, but requires a Schema to achieve this^8^. Similarly, a recently proposed compression algorithm for FASTQ files achieved a very high compression ratio by considering the format^18^. Here, we propose a method which is independent of the underlying data.

Completely gzipped files cannot be easily parsed while streaming. It is necessary to buffer chunks of the compressed data, decompress the chunks and parse them, and put them in relation with previously decompressed chunks. While this approach is simpler when using FASTQ files, it breaks the structure of XML files and loses any benefit. Metadata stored in BON can be parsed during the transfer while storing compressed large biological data reduces the file size. While this results in larger file sizes than fully compressed files, it is still able to be reduce the file size up to 80%.

In sum, BON is a lean file format for almost all biological data based on a widely used notation and is more stable than other available formats. BON enables a clear structuring of biological data and the accompanying metadata using the JSON notation. We demonstrate this versatility with our basic BON library that can be used for all data sets by adjusting the basic BON object without the need to adjust the underlying parser. We revealed a data compression of up to 70% on sequence data and up to 87% on phylogenetic data, depending on the number and size of the compressed data. This compression is especially powerful when used with large data sets containing metadata that are increasingly common in biology.

## Methods

### Data set preparation

All data sets were obtained from NCBI (https://www.ncbi.nlm.nih.gov). FASTQ data sets were downloaded and created using the NCBI SRA (Short Read Archive) toolkit version 2.8.2 (https://github.com/ncbi/sra-tools). XML data sets in the TinySeq format were downloaded from NCBI using the Entrez REST API^19^. All accession numbers can be found in the git repository under “accessions”.

### Genome data sets

Genome sequences were assembled from the RefSeq NCBI database using the GenBank accession numbers in the assembly reports at ftp://ftp.ncbi.nlm.nih.gov/genomes/refseq/assembly_summary_refseq.txt. Only assembled chromosome or genomes were selected. The genome data set comprised the following subsets:

### Collection data sets

The collection data set comprises three subsets; (i) virus genomes (“viruses”), (ii) expressed sequence tags from plants (“Plant EST”), and (iii) the *Blumeria graminis* genome scaffold sequences (“Bgram scaffolds”). Viral genome sequences were downloaded using the accession derived from the search term “(viruses [organism] NOT Bacteria [organism]) AND “complete genome” [title]” in the NCBI RefSeq database. Plant expressed sequence tags (ESTs) were downloaded using 1,000,000 random accession derived from the first 2,000,000 results for the search term “viridiplantae[organism]” in the NCBI EST database. The *Blumeria graminis* genome scaffolds were downloaded directly from NCBI (GenBank accession GCA 000151065.3).

### Protein data sets

We created two subsets of plant and human protein sequences. Plant protein sequences were downloaded using search results for the term “viridiplantae[organism] NOT (putative OR hypothetical OR predicted)” in the NCBI protein database, which resulted in 627,778 protein sequences. For the human protein data set, the first million reads for the search term “homo sapiens [organism] NOT (putative OR hypothetical OR predicted)” were downloaded.

### FASTQ data sets

To analyse the impact of sequence length and quality on the compression, NCBI SRA archives were screened for runs with short reads, long reads, and a mixture of short and long reads. SRA accession numbers ERR1662731, ERR1988801, SRR390728, SRR5710238, SRR5787977, and SRR5802898 were downloaded using the NCBI SRA toolkit. From each run the first million FASTQ entries were extracted, apart from run ERR1662731 which comprised 767,298 FASTQ entries.

### Phylogenetic data sets

Phylogenetic data sets were downloaded as Nexus files from Treebase (https://treebase.org/treebase-web/home.html) and converted into NeXML^6^.

### Calculating file sizes

For each data subset the file sizes were calculated in bytes. XML files were analysed as coming directly from Entrez and therefore contained line breaks (“*\*n”). The completely compressed files were created using the same input files and zlib settings (compression level 6). To verify the file sizes obtained from our analysis pipeline, a random sub sets form each data set was compressed using gzip v 1.6 (ftp://ftp.gnu.org/gnu/gzip/) using following command: gzip -c -6 dataset | wc -c. The analysis pipeline can be found in the repository in tools/datasampler/src.

### Software

The BON implementation, documentation and examples are available at https://gitlab.com/janpb/bon. The Bon class in bon.py implements the basic BON class, the BonObject class in bon_object.py implements a basic bon object, and the BonReader class in reader.py implements a basic BON reader. We implemented BON converters for the formats analysed which can be found in the converter directory. This also provides examples on how to include BON in existing pipelines and applications.

### XML and JSON parser complexity analysis

We used cloc 1.70 (https://github.com/AlDanial/cloc) to count the lines of code and dependent files in the corresponding source files. Files with the following regular expression pattern were excluded from the analysis: .*test.*|tests|.*doc.*|.*tutorial.*|examples.

### Analysis

The analysis described here was performed using a 64 bit Linux Desktop with an Intel i5–4670 CPU (4 cores at 3.40 GHz) and 32 GB of RAM running Ubuntu 17.04. All programs were written in Python 3.5.3. XML and JSON data were parsed using the internal Python modules xml.etree.ElementTree and json, respectively. The biological data was compressed using the internal Python modules zlib (compression level 6) and base64. Phylogenetic data was parsed using Bio.Phylo^20^. Figure 3 was prepared using matplotlib^21^ (Table 1).

**Table 1:** Genome accessions used in the Genome data set.

Organism	Subset name	GenBank accession
*Brachypodium distachyon*	Bdist	GCA 000005505.2
*Drosophila melanogaster*	Dmela	GCA 000001215.4
*Escherichia coli*	Ecoli	GCA000005845.2
*Homo sapiens*	Hsapi	GCA 000001405.24
*Mus musculus*	Mmus	GCA 000001635.7
*Saccharomyces cerevisiae*	Scere	GCA 001051215.1
*Zea Mays*	Zmays	GCA 001644905.1

Subset name indicates the used abbreviation used in this analysis.

## Electronic supplementary material

Supplementary Information
