# Supplementary material for: The Biological Object Notation (BON): a structured file format for biological data
Source: Sci Rep. 2018 Jun 25;8:9644. doi: 10.1038/s41598-018-28016-6 (PMC6018389; doi:10.1038/s41598-018-28016-6)
Supplement: Supplementary file 1 — Supplementary Information [file 41598_2018_28016_MOESM1_ESM.pdf]

The Biological Object Notation (BON): a structured file format for biological data

Supplementary Material

Jan P. Buchmann, Mathieu Fourment, Edward C. Holmes

Supplementary Note 1

Counting the lines of code (LOC) within the source code of a program can be used to roughly describe its complexity: the greater LOC, the more complexity can be assumed. We are aware that using LOC as a metric for complexity when comparing different programming languages is problematic. However, when compared the same programming language, the verbosity of a programming language can be neglected.

We compared the most widely used open source JSON and XML parsers(Supplementary Table 1). With the exception of rapidxml, all XML parsers have more dependent files and line of codes. However, rapidxml is not fully W3C compliant and therefore considered a special type of XML parser.

Supplementary Note Table 1: Summary of common JSON and XML parsers available in the Python and C++ programming languages. The number of files (Files) and lines of code (LOC) are considered proxies for the complexity of the parser. Blank lines and comments have been excluded from the count.

Format	Library	Language	Version	Files	LOC
JSON	jsoncpp	C++	1.7.2	15	5134
	json	Python	3.5	5	680
XML	Xerces	C/C++	3.1.3	866	160157
	rapidxml	C++	1.13	6	2580
	xml	Python	3.5	22	5117

Supplementary Note 2

Comparison of clutter between TinySeq and BON. TinySeq represent a typical XML file as received using NCBI's EDirect utility (https://www.ncbi.nlm.nih.gov/books/NBK179288).

```
1  <?xml version="1.0" ?>\n
2  <!DOCTYPE TSeqSet PUBLIC "-//NCBI//NCBI TSeq/EN" "https://www.ncbi.nlm.nih.gov/dtd/NCBI_TSeq.dtd">\n
3  <TSeqSet><TSeq>\n
4  ␣␣<TSeq_seqtype␣value="protein"/>\n
5  ␣␣<TSeq_accver>AP_000196.1</TSeq_accver>\n
6  ␣␣<TSeq_taxid>10515</TSeq_taxid>\n
7  ␣␣<TSeq_orgname>Human adenovirus 2</TSeq_orgname>\n
8  ␣␣<TSeq_defline>E4 ORF1 [Human adenovirus 2]</TSeq_defline>\n
9  ␣␣<TSeq_length>128</TSeq_length>\n
10 ␣␣<TSeq_sequence>MAAAVEA...</TSeq_sequence>\n
11 </TSeqSet></TSeq>\n
```

Listing 1: TinySeq XML example from NCBI's EDirect servers. Lines 1 and 2 are considered the header. Line breaks are indicated as '\n' and space characters as ␣. Characters in red are considered part of the syntax while black characters part of the data. Characters indicated in gray and spaces (␣) were excluded in calculating clutter.

```
1 {"size":1,"data":{"defline":0,"typ":0,"taxid":0,"length":0,"orgname":0,"sequence":0,"accver":0}}\n
2 [{"seqtype":"protein",\n
3 ␣␣"accver":"AP_000196.1",\n
4 ␣␣"taxid":"10515",\n
5 ␣␣"orgname":"Human adenovirus 2",\n
6 ␣␣"defline":"E4 ORF1 [Human adenovirus 2]",\n
7 ␣␣"length":"128",\n
8 ␣␣"sequence":"MAAAVEA..."}]\n
```

Listing 2: BON equivalent of a TinySeq XML entry. Lines 1 represents the header. Line breaks are indicated as '\n' and space characters as ␣. Characters in red are considered part of the syntax while black characters part of the data. Characters indicated in gray and spaces (␣) were excluded in calculating clutter.

Supplementary Note Table 2: Clutter calculations for NCBI accession AP_000196.1 as shown in Listings 1 (TinySeq XML) and 2 (BON). The sequence data has been excluded. Header indicates the amount of bytes required for the header. Syntax indicates the amount of bytes required by the format syntax. Data indicates the amount of bytes required for the biological data.

Format	Header [bytes]	Data [bytes]	Syntax [bytes]	Total [bytes]
TinySeq XML	120	80	159	359
BON	96	80	93	269

Supplementary Note 3

This section shows examples of possible pitfalls when parsing meta data from the unstructured file formats FASTA and FASTQ. While in most cases the parsers will not fail, they can introduce errors as they parse the wrong data, e.g. a functional description can be parsed as host. Correcting those errors requires to i) check every entry; ii) perform additional checks which subsequently increase the complexity of the parser, thereby increasing the change to introduce additional errors.

3.1 FASTA files

We present two examples where the header line of a FASTA file contains different number of columns when split by a specific delimiter.

Example 1 The Supplementary Material file GB_trnHpsbA.fa from Spouge and Mariño-Ramírez (2012) contains 4716 FASTA sequences. The FASTA headers are delimited by an underscore (_), e.g. `>Acacia_oswaldii_10880561.0.0`. However, some entries are formatted as follows: `>Acorus_sp._'Mt._Emei'_62903272.0.0`. Splitting the header lines using the underscore as delimiter results between 3 and 8 fields (Supplementary Note Table 3).

Supplementary Note Table 3: Number of fields in FASTA headers in the Supplementary Material of Spouge and Mariño-Ramírez (2012) when using '_' as delimiter.

entries	fields
4622	3
34	4
27	5
12	6
14	7
7	8

The histogram was created using following command:

```
grep "^>" GB_trnHpsbA.fa | awk -F_ '{print NF}' | sort -n | uniq -c
```

Example 2 Supplementary Material S1 from Glaser *et al.* (2013) contains 179 predicted proteins in FASTA format. Examples for FASTA headers are `>SnonIR41a` or `>Snon-Fatty-acyl CoA reductase 1`. Using '-' as delimiter results between one and four fields (Supplementary Note Table 4).

Supplementary Note Table 4: Number of fields in FASTA headers in Supplementary Material S1 from Glaser *et al.* (2013) when using '-' as delimiter.

entries	fields
145	1
8	2
21	3
5	4

The histogram was created using following command:

```
pdftotext Glaser2013_suppl.pdf - | grep "^>"  | awk -F- '{print NF}' | sort -n | uniq -c
```

3.2 FASTQ files

The FASTQ header is indicated by an "@". FASTQ is an ambiguous format because the character indicating the start of the first and third part of a FASTQ entry can occur at the beginning of the quality string as well. This are the characters "@" and "+", respectively. While in most cases each part of a FASTQ file starts on a new line, it is allowed to have line breaks in the sequence (Cock *et al.*, 2010). We tested how often an '@' or '+' appear at the start of a quality string to indicate the ambiguity and possible pitfalls when parsing FASTQ files (Supplementary Note Table 5). In addition, the title of a FASTQ entry can have similar problems as the FASTA headers described in Example 2.

Supplementary Note Table 5: Summary of the occurrence of the ambiguous characters '@' and '+' in FASTQ SRA archives. Total entries described the number of FASTQ entries in the archive. Delimiter indicates the occurrence of the corresponding delimiter at the start of a quality string.

SRA Accession	Total entries	Delimiter	
		@	+
ERR1662731	767,298	3,332	0
ERR1988801	10,393,547	0	0
SRR390728	7,178,576	0	1,178
SRR5710238	40,921,608	18,222,425	42,529
SRR5787977	26,453,754	0	0
SRR5802898	21,281,470	105,603	0

The occurrences were counted using following commands:

```
sed -n '0~4p' fastqfile | grep -c  "^@"
sed -n '0~4p' fastqfile | grep -c  "^+"
```

Supplementary Tables

Supplementary Table 1: Summary of file sizes for nucleotide sequences in TinySeq XML, BON and compressed BON. Mbp: Megabasepairs; MiB: mebibytes (2^{20} bytes); Data sets: name of data set analysed, Subset: name of subset, Size: number of sequence in subset; Attrib: size of uncompressed (zlib-) and compressed (zlib+) attributes in MiB; Data size: For each subset the shortest (Min), longest (Max), average (Avg) and total (Total) length in Mbp is given. XML: TinySeq File size (sF) uncompressed (zlib-) and compressed (zlib+), its ratio to uncompressed attribute data (rD); BON: File size (sF) of uncompressed BON and its ratios to the TinySeq file size (rF) and to its corresponding uncompressed attribute data (rD); cBON: file size of the compressed BON and its ratio to TinySeq (rF) and to its corresponding compressed attribute data (rD).

Dataset			Attrib [MiB]		Data size [Mbp]				XML sF [MiB]				BON			cBON		
Name	Subset	Size	zlib-	zlib+	Min	Max	Avg	Total	zlib-	zlib+	rF	rD	sF [MiB]	rD	rF	sF [MiB]	rF	rD
Genomes	Dmela	7	131.18	48.79	1.35	32.08	19.65	137.55	131.18	36.59	0.27	1.00	131.18	1.00	1.00	48.79	0.37	1.00
	Ecoli	1	4.43	1.71	4.64	4.64	4.64	4.64	4.43	1.28	0.28	1.00	4.43	1.00	1.00	1.71	0.39	1.00
	Bdist	5	259.19	98.74	28.56	75.12	54.36	271.78	259.19	74.06	0.28	1.00	259.19	1.00	1.00	98.75	0.38	1.00
	Hsapi	25	2945.22	1032.15	0.17	248.96	123.53	3088.29	2945.23	774.14	0.26	1.00	2945.23	1.00	1.00	1032.15	0.35	1.00
	Mmusc	21	2599.26	947.62	61.43	195.47	129.79	2725.52	2599.27	710.76	0.27	1.00	2599.26	1.00	1.00	947.62	0.36	1.00
	Scere	17	11.53	4.46	0.08	1.53	0.71	12.09	11.53	3.35	0.29	1.00	11.53	1.00	1.00	4.46	0.39	1.00
	Zmays	10	2078.13	710.00	154.59	318.11	217.91	2179.08	2078.13	532.50	0.25	1.00	2078.13	1.00	1.00	710.00	0.34	1.00
Collection	Plant EST	10^6	706.53	447.53	>0.00	>0.01	>0.01	544.35	970.91	197.25	0.20	0.73	803.81	0.83	0.88	544.81	0.56	0.82
	Virus genomes	66725	1230.82	490.02	0.02	2.24	>0.01	1282.02	1246.96	308.95	0.24	0.99	1237.21	0.99	0.99	496.40	0.40	0.99
	Bgrami contigs	6843	114.34	34.85	0.67	9.69	>0.01	118.74	115.97	25.26	0.21	0.99	115.00	0.99	0.99	35.50	0.31	0.98

Supplementary Table 2: Summary of file sizes for protein sequences in TinySeq XML, BON and compressed BON. aa: amino acids; MiB: mebibytes (2^{20} bytes); Data sets: name of data set analysed, Subset: name of subset, Size: number of sequence in subset; Attrib: size of uncompressed (zlib-) and compressed (zlib+)attributes in MiB; Data size: For each subset the shortest (Min), longest (Max), average (Acg) and total (Total) length in aa is given. XML: TinySeq File size (Fs) and its ratio to uncompressed attribute data (rD); BON: File size of uncompressed BON and its ratios to the TinySeq file size (rF) and to its corresponding uncompressed attribute data (rD); cBON: file size of the compressed BON and its ratio to TinySeq (rF) and to its corresponding compressed attribute data (rD).

Dataset			Attrib [MiB]		Data size [aa]				XML				BON			cBON		
									sF [MiB]									
Name	Subset	Size	zlib-	zlib+	Min	Max	Avg	Total	zlib-	zlib+	rF	rD	Fs [MiB]	rD	rF	Fs [MiB]	rF	rD
Protein	Hsapi	10^6	424.82	370.04	1	35991	336.82	336817042	664.46	81.37	0.12	0.64	520.32	0.78	0.82	465.54	0.70	0.79
	Plant	627778	302.80	263.73	1	21004	371.08	232954947	461.13	137.52	0.29	0.66	363.36	0.79	0.83	324.29	0.70	0.81

Supplementary Table 3: Summary of file sizes for nucleotide sequences in FASTQ, BON and compressed BON. bp: basepairs; MiB: mebibytes (220 bytes); Data sets: name of data set analysed, Subset: name of subset, Size: number of sequence in subset; Attrib: size of uncompressed (zlib-) and compressed (zlib+) attributes in MiB; Data size: For each subset the shortest (Min), longest (Max), average (Avg) and total (Total) length in aa is given. FASTQ: FASTQ file size (sF) and its ratio to uncompressed attribute data (rD); BON: File size of uncompressed BON and its ratios to the FASTQ file size (rF) and to its corresponding uncompressed attribute data (rD); cBON: File size of the compressed BON and its ratio to FASTQ (rF) and to its corresponding compressed attribute data (rD).

Dataset			Attrib [MiB]		Data size [bp]				FASTQ				BON			cBON		
									sF [MiB]									
Name	Subset	Size	zlib-	zlib+	Min	Max	Avg	Total	zlib-	zlib+	rF	rD	sF [MiB]	rD	rF	sF [MiB]	rF	rD
SRA	ERR1662731	767298	320.89	215.47	151	251	202.38	155287898	363.15	183.67	0.50	0.88	366.99	1.01	0.87	261.57	0.72	0.82
	ERR1988801	10^6	120.90	111.80	51	51	51.00	51000000	167.42	110.68	0.66	0.72	180.99	1.08	0.67	171.88	1.03	0.65
	SRR390728	10^6	160.01	132.95	72	72	72.00	72000000	205.57	126.34	0.61	0.78	220.09	1.07	0.73	193.04	0.94	0.69
	SRR5710238	10^6	409.87	312.41	202	202	202.00	202000000	457.34	261.39	0.57	0.90	469.95	1.03	0.87	372.50	0.81	0.84
	SRR5787977	10^6	596.79	297.64	300	300	300.00	300000000	644.26	250.30	0.38	0.93	656.87	1.02	0.91	357.72	0.56	0.83
	SRR5802898	10^6	150.08	138.15	50	50	50.00	50000000	227.68	137.73	0.60	0.66	210.16	0.92	0.71	198.23	0.87	0.70

Supplementary Table 4: Summary of file sizes for phylogenetic data in NeXML, BON and compressed BON. Nodes: phylogenetic internal and leaf nodes; MiB: mebibytes (220 bytes); Data sets: name of data set analysed, Subset: name of subset, Size: number of sequence in subset; Attrib: size of uncompressed (zlib-) and compressed (zlib+) attributes in MiB; Data size: For each subset the smallest (Min), largest (Max), average (Avg) and total (Total) number of phylogenetic tree nodes is given. Nexml: NeXML file size (sF) and its ratio to uncompressed attribute data (rD); BON: File size of uncompressed BON and its ratios to the NeXML file size (rF) and to its corresponding uncompressed attribute data (rD); cBON: File size of the compressed BON and its ratio to NeXML (rF) and to its corresponding compressed attribute data (rD).

Dataset			Attrib [MiB]			Data size [bp]			NeXML sF [MiB]			BON			cBON			
Name	Subset	Size	zlib-	zlib+	Min	Max	Avg	Total	zlib-	zlib+	rF	rD	sF [MiB]	rD	rF	sF [MiB]	rF	rD
Phylo	treebase0	60	0.04	0.02	5	7	6.75	405	0.06	>0.01	0.13	0.62	0.04	0.69	0.90	0.02	0.34	0.80
	treebase1	5	0.13	0.03	144	261	220.00	1100	0.21	0.03	0.14	0.63	0.13	0.63	1.00	0.03	0.12	0.98
	treebase2	541	32.37	6.81	209	799	494.05	267281	50.56	7.29	0.14	0.64	32.42	0.64	1.00	6.85	0.14	0.99

References

Cock, P.J.A., **Fields, C.J.**, **Goto, N.**, **Heuer, M.L.** and **Rice, P.M.** (2010) The Sanger FASTQ file format for sequences with quality scores, and the Solexa/Illumina FASTQ variants. *Nucleic Acids Research*, **38**, 1767–1771.

Glaser, N., **Gallot, A.**, **Legeai, F.**, **Montagné, N.**, **Poivet, E.**, **Harry, M.**, **Calatayud, P.A.** and **Jacquin-Joly, E.** (2013) Candidate chemosensory genes in the Stemborer Sesamia nonagrioides. *International journal of biological sciences*, **9**, 481–495.

Spouge, J.L. and **Mariño-Ramírez, L.** (2012) The practical evaluation of DNA barcode efficacy. *Methods in molecular biology (Clifton, N.J.)*, **858**, 365–377.
